# The Mediating Effect of Risk Awareness on the Relationship Between Infectious Disease Knowledge and Protective Behaviors Among Earthquake-Affected Populations

**DOI:** 10.3390/healthcare14172688

**Published:** 2026-08-24

**Authors:** Selda Seçginli, Nesrin İlhan, Gizemnur Torun, Merve Altıner Yaş, Seda Doğru Bolat

**Affiliations:** 1 Department of Nursing, Faculty of Health Sciences, Atlas University, Istanbul 34408, Türkiye; 2 Department of Nursing, Faculty of Health Science, Istanbul Medeniyet University, Istanbul 34700, Türkiye; nesrin.ilhan@medeniyet.edu.tr; 3 Department of Nursing, Faculty of Health Science, Kocaeli University, Kocaeli 41380, Türkiye; gizemnur.torun@kocaeli.edu.tr; 4 Public Health Nursing Department, Faculty of Nursing, Istanbul University, Istanbul 34116, Türkiye; merve.altiner@istanbul.edu.tr; 5 Family Health Unit No. 09, Family Health Center No. 03, Hatay 31800, Türkiye; sedadogru12345@gmail.com

**Keywords:** infectious diseases, infectious disease knowledge, risk awareness, protective behaviors, earthquakes

## Abstract

**Highlights:**

**What are the main findings?**

- Greater infectious disease knowledge was associated with higher communicable disease risk awareness and stronger protective and handwashing behaviors among earthquake-affected individuals.
- Self-protection awareness and personal contact awareness significantly mediated the relationship between infectious disease knowledge and protective and handwashing behaviors.

**What are the implications of the main findings?**

- Public health interventions in long-term post-disaster settings should incorporate strategies to improve infectious disease knowledge and risk awareness to support protective and handwashing behaviors.
- Educational interventions that enhance self-protection and personal contact awareness may increase adherence to infection prevention behaviors in disaster-affected communities.

**Abstract:**

**Background:** Long-term post-disaster settings increase infectious disease risks, requiring adequate knowledge and risk awareness to promote protective behaviors. This study was conducted to investigate the relationships between infectious disease knowledge, risk awareness, and protective behaviors among earthquake-affected individuals, and to test the mediating effect of risk awareness on these relationships. **Methods**: This cross-sectional study was conducted among 257 participants living in two container settlements in an earthquake-affected region. Data were collected with the Sociodemographic Information Form, the Infectious Disease Knowledge Questionnaire (IDKQ), and the Communicable Diseases Risk Awareness and Protection Scale (CDRAPS). Data were analyzed using descriptive statistics, *t*-tests, ANOVA, Pearson’s correlation analysis, and the mediating effect of risk awareness was tested using Hayes’ PROCESS macro (Model 4). **Results:** The mean scores of the IDKQ and CDRAPS were 8.69 ± 5.82 and 131.50 ± 24.49, respectively. Significant positive correlations were found between IDKQ scores and CDRAPS total and subdimension scores (*p* < 0.001). Self-Protection Awareness significantly mediated the relationship between infectious disease knowledge and both Protection Behaviors (β = 0.420, 95% CI [0.299–0.557]) and Handwashing Behaviors (β = 0.104, 95% CI [0.050–0.161]). Personal Contact Awareness significantly mediated the relationship between infectious disease knowledge and Protection Behaviors (β = 0.256, 95% CI [0.117–0.388]) as well as Handwashing Behaviors (β = 0.172, 95% CI [0.118–0.221]). **Conclusions:** Greater infectious disease knowledge was associated with higher protective behaviors, and this association was partially mediated by self-protection awareness and personal contact awareness. Interventions aimed at improving infectious disease knowledge and awareness may contribute to strengthening individuals’ protective and handwashing behaviors.

## 1. Introduction

Infectious diseases remain a major public health concern worldwide, particularly in disaster-affected settings [1,2]. Following earthquakes, conditions such as population displacement, overcrowding, inadequate sanitation, and limited access to healthcare significantly increase the risk of infectious disease outbreaks [1,3,4]. These outbreaks can further intensify the health burden of disasters, complicate emergency response efforts, and delay community recovery [5].

Türkiye is in one of the world’s most seismically active regions, where devastating earthquakes occur frequently. On 6 February 2023, two major earthquakes struck southeastern Türkiye, causing approximately 51,000 deaths and the destruction of about 38,000 buildings [6]. Beyond their immediate impact, earthquakes can substantially affect population health through displacement, poor living conditions, inadequate sanitation, and limited access to healthcare services [7]. In the aftermath of the 2023 earthquakes, prolonged shelter shortages, overcrowded tent settlements, and inadequate sanitation emerged as key risk factors for infectious disease transmission [8].

Evidence suggests that both endemic and non-endemic infectious diseases may emerge following earthquakes [8,9,10]. Post-disaster settings are associated with increased risks of wound-related, waterborne, foodborne, vector-borne, and respiratory infections, posing substantial public health challenges [3,4,10]. A recent meta-analysis reported that the incidence of infectious diseases increases by approximately 1.56 times after earthquakes [11].

Effective prevention and control of infectious diseases, as recommended by the World Health Organization (WHO), require integrated public health measures, including infection prevention and control practices, safe water, sanitation and hygiene services, environmental health interventions, disease surveillance, and timely response strategies to limit the spread of infections [12]. However, the effectiveness of these interventions depends not only on resource availability but also on individuals’ knowledge and understanding of infectious diseases, including their modes of transmission and preventive measures [13]. Infectious disease knowledge enables individuals to recognize health threats, make informed decisions, and adopt appropriate preventive behaviors. In disaster settings, where transmission risks are heightened, increasing knowledge and awareness may play a critical role in promoting protective behaviors and reducing vulnerability to outbreaks [14].

In addition to knowledge, risk awareness plays a crucial role in shaping protective health behaviors. Risk awareness refers to individuals’ perception of the likelihood and severity of a health threat [15]. Individuals with higher risk awareness are more likely to engage in preventive practices such as maintaining hygiene, consuming safe food and water, vaccination uptake, and adherence to public health recommendations. Previous studies have demonstrated consistent associations among knowledge, risk awareness, and preventive behaviors across infectious disease contexts [15,16,17,18]. Emerging evidence suggests that risk awareness may mediate the relationship between knowledge and behavioral responses. For example, Jung and Song reported that risk awareness mediated the relationship between e-health literacy and infectious disease prevention behaviors among young adults [19]. Similarly, Yan et al. found that both knowledge and risk awareness were important predictors of vulnerability to dengue fever following climate-related disasters [20]. Collectively, these findings indicate that greater infectious disease knowledge may enhance risk awareness, thereby promoting protective behaviors.

Despite growing evidence, research on infectious diseases in disaster contexts has primarily focused on epidemiological and environmental determinants, with limited attention to behavioral mechanisms at the individual level. Existing studies highlight increased outbreak risks after earthquakes due to disrupted sanitation and weakened health systems [1,2,4,5], but provide limited insight into how knowledge translates into protective behaviors. Therefore, this study investigates the relationships among infectious disease knowledge, risk awareness, and protective behaviors, and examines whether risk awareness mediates this relationship in earthquake-affected populations. Understanding these pathways may inform targeted health education and disaster preparedness interventions to reduce infectious disease risks and strengthen community resilience. This study was conducted to address research questions and examined the levels of infectious disease knowledge, risk awareness, and protective behaviors among earthquake-affected individuals, their variation according to sociodemographic and health-related characteristics, and the mediating role of risk awareness in the relationship between infectious disease knowledge and protective behaviors.

## 2. Materials and Methods

### 2.1. Study Design

This study was conducted within the framework of a project aimed at improving knowledge, awareness, and preventive practices regarding infectious diseases in earthquake-affected regions. It was designed as a cross-sectional study using mediation analysis to examine the relationships among infectious disease knowledge, risk awareness, and protective behaviors. The study was reported in accordance with the STROBE checklist for cross-sectional studies. Data were collected between October and December 2025 in two container settlements in Hatay, Türkiye. The study population consisted of 826 individuals aged 18 years and older. The required sample size was calculated as 204 based on a 90% confidence level and a 5% margin of error (N = 826, Z = 1.645, *p* = 0.5, d = 0.05), with a finite population correction applied to account for the finite study population. Participants were recruited using convenience sampling, and 257 eligible individuals voluntarily participated in the study, exceeding the minimum required sample size. Inclusion criteria were being 18 years and older, residing in a container settlement, being literate, and owning a smartphone.

### 2.2. Data Collection

Data were collected using an online self-administered questionnaire developed in Google Forms^®^ and distributed via WhatsApp groups in container settlements. After providing informed consent by selecting “I agree,” participants accessed the survey items. The questionnaire took approximately 10–12 min to complete.

### 2.3. Data Collection Tools

Data were collected using a Sociodemographic Information Form, the Infectious Disease Knowledge Questionnaire (IDKQ), and the Communicable Diseases Risk Awareness and Protection Scale (CDRAPS).

#### 2.3.1. Sociodemographic Information Form

The sociodemographic information form included 11 items assessing participants’ sociodemographic and health-related characteristics, including age, sex, education, marital and employment status, income, perceived health status, chronic disease, medication use, post-earthquake infectious disease history, and willingness to participate in an online training program.

#### 2.3.2. Infectious Disease Knowledge Questionnaire (IDKQ)

The Infectious Disease Knowledge Questionnaire (IDKQ), developed by Seçginli et al. [21], is a 17-item scale assessing knowledge of infectious diseases. It includes four subdimensions: infectious diseases and misinformation (Items 1–5), personal and environmental hygiene (Items 6–10), immunization and personal protection (Items 11–13), and water- and vector-borne diseases (Items 14–17). Correct responses were scored as 1, whereas incorrect and “I don’t know” responses were scored as 0; some items were reverse-coded. Total scores range from 0 to 17, with higher scores indicating greater knowledge. In the original study, KR-20 was 0.735 [21]. In the present study, KR-20 was 0.94 for the total scale and 0.75–0.93 for the subdimensions.

#### 2.3.3. Communicable Diseases Risk Awareness and Protection Scale (CDRAPS)

The Communicable Diseases Risk Awareness and Protection Scale (CDRAPS), developed by Ener, Seyfeliand Çetinkaya [22], is a 36-item, six-factor scale measuring risk awareness and protective behaviors related to communicable diseases. The subdimensions include common life risk awareness, self-protection awareness, protection behaviors, handwashing behaviors, social protection awareness, and personal contact awareness. Items are rated on a 5-point Likert scale, with awareness items ranging from “Strongly Disagree” to “Strongly Agree” and behavioral items from “Never” to “Always.” The total score ranges from 36 to 180, with higher scores indicating higher awareness and protective behaviors [22]. In the original study, Cronbach’s alpha was 0.91 (subscales: 0.60–0.78). In the present study, Cronbach’s alpha was 0.98 (subscales: 0.70–0.95).

### 2.4. Ethical Considerations

Ethical approval was obtained from the Atlas University Non-Interventional Research Ethics Committee (Date: 21 August 2024; Approval No:07/05). Participants were informed about the study purpose, confidentiality, and voluntary participation, and online informed consent was obtained prior to data collection.

### 2.5. Statistical Analysis

Statistical analysis was performed using IBM SPSS Statistics for Windows (version 25.0, IBM Corp., Armonk, NY, USA) and PROCESS Macro 4.2 [23]. Normality was evaluated through skewness and kurtosis coefficients. As the values were generally within ±1.5 and all fell within the acceptable ±2.0 range, the distributions were considered approximately normal [24]. Descriptive statistics were used to summarize the study variables, and Pearson correlation analysis was performed to examine the relationships among infectious disease knowledge, risk awareness, and protection behaviors. For comparisons between two groups, independent-samples *t*-tests were used. For comparisons involving more than two groups, the homogeneity of variances was assessed using Levene’s test. When the assumption was met (*p* ≥ 0.05), one-way ANOVA with Tukey HSD post hoc tests was used; when it was violated (*p* < 0.05), Welch’s ANOVA with Games–Howell post hoc tests was used.

To investigate the mechanisms underlying these relationships, mediation analysis was conducted using Hayes’ PROCESS macro (Model 4) with 5000 bootstrap samples and 95% confidence intervals. Infectious disease knowledge was entered as the independent variable, while protection behaviors and handwashing behaviors were analyzed separately as dependent variables. The four risk awareness dimensions—Common Life Risk Awareness, Self-Protection Awareness, Social Protection Awareness, and Personal Contact Awareness—were specified as parallel mediators. This approach enabled the examination of direct, indirect, and total effects while accounting for the simultaneous contribution of multiple mediating pathways. Multicollinearity among the variables included in the mediation models was assessed using variance inflation factor (VIF) and tolerance statistics [25]. In addition to the parallel mediation models, separate single-mediator models for each awareness subdimension were examined as sensitivity analyses to evaluate the robustness of the individual indirect pathways without simultaneously including the correlated mediators in the same outcome equation. Indirect effects were considered statistically significant when the bootstrap 95% confidence interval did not include zero [23]. For all other statistical tests, statistical significance was set at *p* < 0.05.

## 3. Results

### 3.1. Descriptive Characteristics

The descriptive characteristics of the participants are presented in Table 1. The mean age of the participants was 47.14 ± 14.63 years, and 62.3% were female (Table 1).

### 3.2. Associations Between IDKQ and CDRAPS

Mean IDKQ and CDRAPS scores are presented in Table 2. IDKQ was significantly and positively correlated with all CDRAPS subdimensions (*p* < 0.01), including common life risk awareness (r = 0.729), self-protection awareness (r = 0.835), protection behaviors (r = 0.896), handwashing behaviors (r = 0.843), social protection awareness (r = 0.481), personal contact awareness (r = 0.843), and the total CDRAPS score (r = 0.877).

### 3.3. Comparison of IDKQ and CDRAPS Scores by Sociodemographic and Health Characteristics

Participants aged 18–44 years had significantly higher IDKQ and CDRAPS scores compared to older age groups (*p* < 0.001). Similarly, university graduates showed higher scores than those with lower educational levels, with a clear gradient across education categories (*p* < 0.001). Single participants had higher IDKQ and CDRAPS scores than married and widowed/divorced individuals (*p* < 0.001). Employed participants also demonstrated higher scores than unemployed participants (IDKQ = *p* < 0.001; CDRAPS = *p* < 0.05). Participants reporting good/very good health had higher IDKQ scores than those with poorer perceived health (*p* < 0.05). Individuals without chronic disease (*p* < 0.001) and those not using regular medication (IDKQ: *p* < 0.01; CDRAPS: *p* < 0.05) had significantly higher scores. Participants who did not wish to participate in infectious disease education had significantly higher IDKQ and CDRAPS scores than those who were willing to participate (*p* < 0.001) (Table 1).

### 3.4. Mediation Analysis of Awareness Subdimensions

Prior to interpretation of the parallel mediation models, multicollinearity among the predictors entered simultaneously in the outcome regression equations was examined. Tolerance values ranged from 0.147 to 0.586, and VIF values ranged from 1.708 to 6.816. The highest VIF values were observed for Self-Protection Awareness (VIF = 6.816, tolerance = 0.147) and Personal Contact Awareness (VIF = 5.282, tolerance = 0.189), indicating some degree of multicollinearity among the predictors.

In single-mediator models (Supplementary File), all awareness subdimensions were significantly and positively associated with both protection and handwashing behaviors, with significant indirect effects indicating partial mediation.

**Figure 1:**
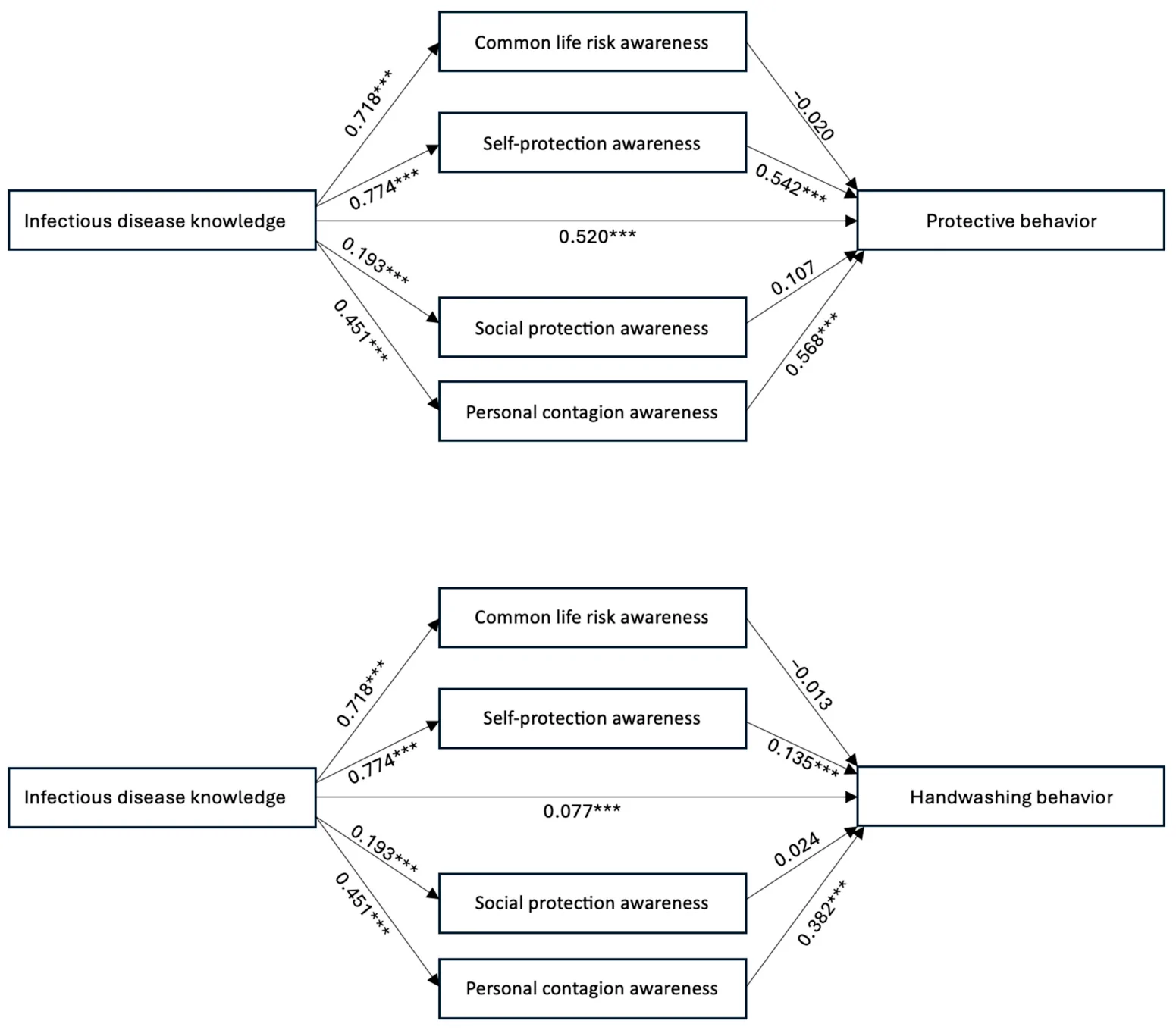
Unstandardized path coefficients for the parallel mediation models predicting protective behaviors and handwashing behaviors. Note. Values represent unstandardized path coefficients (B). The coefficients displayed on the direct paths represent the direct effects of infectious disease knowledge on protective behaviors and handwashing behaviors after controlling for all mediators. *** *p* < 0.001.

To determine the unique contribution of each awareness subdimension, all mediators were entered simultaneously into a parallel mediation model (Figure 1, Table 3). In Model 1 (protection behaviors), the model was significant (R^2^ = 0.893, F = 417.633, *p* < 0.001), explaining 89.3% of the variance. Infectious disease knowledge remained a significant predictor (β = 0.520, *p* < 0.001). Self-protection awareness (β = 0.542, *p* < 0.001) and personal contact awareness (β = 0.568, *p* < 0.001) were significant, whereas common life risk awareness (β = −0.020, *p* = 0.707) and social protection awareness (β = 0.107, *p* = 0.240) were not.

In Model 2 (handwashing behaviors), the model was significant (R^2^ = 0.857, F = 300.526, *p* < 0.001), explaining 85.7% of the variance. Infectious disease knowledge remained significant (β = 0.077, *p* < 0.001). Self-protection awareness (β = 0.135, *p* < 0.001) and personal contact awareness (β = 0.382, *p* < 0.001) were significant predictors, while common life risk awareness (β = −0.013, *p* = 0.505) and social protection awareness (β = 0.024, *p* = 0.449) were not.

As shown in the Supplementary File and Table 3 and Table 4, mediation analyses examined the roles of awareness subdimensions in the relationship between infectious disease knowledge and protection and handwashing behaviors. In single-mediator models (Supplementary File), all awareness subdimensions significantly mediated both outcomes, with bootstrap confidence intervals excluding zero, indicating partial mediation. For protection behaviors (Y_1_), indirect effects were significant for all subdimensions, with the strongest effects observed for self-protection awareness (Effect = 0.576, 95% CI = 0.483–0.669) and personal contact awareness (Effect = 0.513, 95% CI = 0.387–0.640). For handwashing behaviors (Y_2_), all mediators were also significant, with the strongest effects for personal contact awareness (Effect = 0.232, 95% CI = 0.192–0.272) and self-protection awareness (Effect = 0.199, 95% CI = 0.157–0.238). Because direct effects remained significant in all single-mediator models, results supported partial mediation.

To evaluate the unique contribution of each awareness subdimension, a parallel mediation analysis was conducted (Table 4). For protection behaviors (Y_1_), the total effect of infectious disease knowledge was significant (Effect = 1.202, 95% CI = 1.129–1.276, *p* < 0.001). The direct effect also remained significant after including all mediators (Effect = 0.520, 95% CI = 0.410–0.630, *p* < 0.001), as did the total indirect effect (Effect = 0.682, 95% CI = 0.557–0.798). Significant indirect effects were observed only for self-protection awareness (Effect = 0.420, 95% CI = 0.299–0.557) and personal contact awareness (Effect = 0.256, 95%CI = 0.117–0.388), while common life risk awareness (Effect = −0.015, 95% CI = −0.106–0.059) and social protection awareness (Effect = 0.021, 95% CI = −0.013–0.058) were not significant.

For handwashing behaviors (Y_2_), the total effect was significant (Effect = 0.349, 95% CI = 0.322–0.377, *p* < 0.001), as was the direct effect (Effect = 0.077, 95% CI = 0.038–0.117, *p* < 0.001) and the total indirect effect (Effect = 0.272, 95% CI = 0.232–0.309). Only self-protection awareness (Effect = 0.104, 95% CI = 0.050–0.161) and personal contact awareness (Effect = 0.172, 95% CI = 0.118–0.221) showed significant indirect effects, whereas common life risk awareness (Effect = −0.009, 95% CI = −0.041–0.023) and social protection awareness (Effect = 0.005, 95% CI = −0.010–0.021) were not significant. Overall, the results indicated that Self-Protection Awareness and Personal Contact Awareness partially mediated the relationships in both models. These pathways were also significant in the single-mediator sensitivity analyses, whereas Common Life Risk Awareness and Social Protection Awareness showed significant indirect effects only when examined individually, suggesting substantial shared variance among the awareness subdimensions.

## 4. Discussion

This study assessed the levels of infectious disease knowledge, risk awareness, and protective behaviors among people affected by earthquakes; examined the associations among these variables; and determined the mediating role of risk awareness in the relationship between infectious disease knowledge and protective behaviors. The findings demonstrated that greater infectious disease knowledge was associated with higher levels of risk awareness and more frequent engagement in both protection behaviors and handwashing behaviors.

This study revealed that earthquake-affected individuals had a moderate level of infectious disease knowledge. Several studies conducted in disaster-prone and environmentally vulnerable populations have reported insufficient knowledge regarding infectious diseases and climate-sensitive infectious diseases [26,27,28]. Likewise, studies conducted in rural communities have found infectious disease knowledge to be moderate or insufficient [29,30]. In addition, participants reported communicable disease risk awareness scores slightly above the midpoint of the scale, similar to those reported among food service workers [31]. Previous studies conducted among adults, university students, and healthcare personnel have reported moderate to high levels of communicable disease risk awareness and preventive behaviors [32,33,34]. Differences between studies may be related to variations in educational attainment, place of residence, and exposure to infectious disease information. The findings indicate that educational interventions remain necessary to strengthen infectious disease knowledge and further enhance risk awareness among disaster-affected and disaster-prone populations.

Several sociodemographic factors were associated with infectious disease knowledge and protective behaviors, including age, education, marital status, and employment status in the study. Participants aged 18–44 years demonstrated higher levels of knowledge and preventive behaviors than older individuals, consistent with previous studies [35,36]. This may reflect younger adults’ greater access to digital health information and public health messaging [37,38]. Higher educational attainment was also associated with greater knowledge and preventive behaviors, supporting findings from studies conducted in different infectious disease contexts [15,35,39]. These results suggest that education plays an important role in promoting health literacy and encouraging preventive health practices.

In addition to sociodemographic factors, the study also investigated whether infectious disease knowledge was associated with protective health behaviors. In the study, infectious disease knowledge was positively associated with protective behaviors among participants. This result is consistent with several studies conducted in populations under disaster and environmental risk [20,28,39]. For example, a study in cyclone-affected communities in China found that higher knowledge was associated with preventive practices [20]. Similarly, studies conducted in various countries during the COVID-19 pandemic have reported that individuals with higher levels of knowledge about the disease were significantly more likely to adopt protective behaviors [16,18,26]. Likewise, research conducted in populations affected by infectious diseases such as avian influenza, dengue fever, and rabies has shown that increases in knowledge levels are associated with improved preventive behaviors [36,40,41]. In contrast, research examining water, sanitation, and hygiene (WASH) practices in flood-prone areas of the Philippines found that households with adequate knowledge and positive attitudes did not always demonstrate appropriate preventive behaviors. Structural barriers, including inadequate infrastructure and an unreliable water supply, limited the translation of knowledge into practice [28]. Although knowledge is essential for the adoption of preventive behaviors, its translation into action is likely influenced by additional cognitive and contextual factors, such as risk perception and environmental constraints.

The mediation analyses revealed that infectious disease knowledge was associated with protective behaviors both directly and indirectly through risk awareness. Similar findings have been reported in previous studies conducted in the context of COVID-19 and dengue [17,19,20]. In the single-mediator models, all four dimensions of risk awareness—common life risk awareness, self-protection awareness, social protection awareness, and personal contact awareness—showed significant indirect effects. However, when all four dimensions of risk awareness were entered simultaneously into the parallel mediation model, only self-protection awareness and personal contact awareness retained significant indirect effects. The attenuation of the indirect effects of common life risk awareness and social protection awareness in the parallel model may also reflect the substantial shared variance among the awareness subdimensions. Therefore, the significant mediator-specific indirect effects of self-protection awareness and personal contact awareness in the parallel model should be interpreted as unique indirect associations conditional on the other correlated mediators, rather than as evidence that the remaining awareness dimensions are unimportant. The different pattern observed in the parallel mediation model may be related to the level of personal relevance of the risk awareness dimensions. Common life risk awareness and social protection awareness reflect broader, more socially oriented perceptions of infectious disease risk and protection, whereas self-protection awareness and personal contact awareness focus directly on the individual and their own protection from infection. Therefore, these two individually oriented dimensions may be more directly linked to protective behaviors [42]. Conceptually, personal contact awareness may reflect an awareness of personal susceptibility to infection, whereas self-protection awareness may reflect an individual’s perceived capacity to engage in protective actions. These concepts are consistent with behavior change theories emphasizing perceived susceptibility and self-efficacy as important cognitive determinants of preventive behavior [43,44]. Furthermore, individuals who had experienced infection themselves or had family members affected by infectious diseases were more likely to engage in preventive behaviors [41]. Taken together, these findings suggest that personally relevant perceptions of infection risk and self-protection may help explain the observed associations between infectious disease knowledge and protective behaviors.

### Limitations and Future Studies

This study has several limitations. First, its analytical cross-sectional design does not allow causal inferences among infectious disease knowledge, risk awareness, and protective health behaviors, even though mediation analysis was applied. Second, all variables were measured using self-reported data, which may be subject to recall and social desirability bias. Third, the use of a convenience sample limits the generalizability of the findings to all earthquake-affected populations. Fourth, the requirement for participants to own a smartphone may have introduced selection bias by potentially excluding individuals with limited financial resources or limited access to digital technology, which may have resulted in underrepresentation of the most economically disadvantaged residents of the container settlements. Fifth, the nearly three-year interval between the earthquakes and data collection may have introduced recall bias regarding participants’ history of infectious diseases after the earthquake and may limit the ability to distinguish earthquake-related effects from longer-term community behaviors. Sixth, substantial intercorrelations were observed among some CDRAPS awareness subdimensions. Although the original scale development and validation study identified these subdimensions as distinct factors, some awareness and behavioral items are conceptually related, which may partly account for the high intercorrelations observed in the present sample. Collinearity diagnostics indicated some degree of multicollinearity, particularly for Self-Protection Awareness and Personal Contact Awareness. Therefore, mediator-specific coefficients in the parallel mediation models should be interpreted cautiously as unique effects conditional on the other correlated mediators. The single-mediator sensitivity analyses further indicated that some indirect effects observed when mediators were examined individually were not significant when all four awareness subdimensions were entered simultaneously, suggesting substantial shared variance among the awareness dimensions.

## 5. Conclusions

This study examined the relationships among infectious disease knowledge, communicable disease risk awareness, and protective behaviors among earthquake-affected individuals. The findings showed that greater infectious disease knowledge was associated with higher risk awareness and stronger engagement in preventive behaviors. Self-protection awareness and personal contact awareness partially mediated the association between infectious disease knowledge and preventive behaviors. The findings were consistent with modeled indirect pathways linking infectious disease knowledge to preventive behaviors through self-protection awareness and personal contact awareness. Among the risk-awareness dimensions, Self-Protection Awareness and Personal Contact Awareness emerged as the most important mediators.

These findings highlight the potential value of incorporating both knowledge enhancement and risk-awareness components into public health interventions in long-term post-disaster settings. Educational programs that improve understanding of infectious diseases while increasing awareness of personal and transmission-related risks may be particularly effective in promoting preventive behaviors. Furthermore, targeted interventions should prioritize groups with lower levels of infectious disease knowledge, risk awareness, and preventive behaviors, with the aim of strengthening preparedness and resilience in long-term post-disaster settings.

## Figures and Tables

**Table 1:** Comparisons of mean IDKQ and CDRAPS scores according to sociodemographic and health-related characteristics of participants.

Variables		IDKQ				CDRAPS			
	*n* (%)	M ± SD	t/F	*p*	Difference	M ± SD	t/F	*p*	Difference
Age (47.14 ± 14.63)									
18–44 age ^a^	105 (40.9)	11.78 ± 4.72	Welch F (2, 82.546) = 32.847	<0.001 ***	a > b, c	142.82 ± 20.68	Welch F (2, 82.129) = 23.896	<0.001 ***	a > b, c
45–64 age ^b^	122 (47.5)	6.68 ± 5.69				124.49 ± 24.27			
65+ age ^c^	30 (11.7)	6.10 ± 5.10				120.36 ± 22.44			
Sex									
Female	160 (62.3)	8.78 ± 5.67	t = 0.299	0.765		132.57 ± 24.74	t = 0.902	0.368	
Male	97 (37.7)	8.55 ± 6.10				129.73 ± 24.10			
Education status									
Literate ^a^	24 (9.3)	4.41 ± 4.06	Welch F (4, 99.21) = 35.05	<0.001 ***	e > a, b, c, d d > a, b c > a	113.45 ± 13.77	Welch F (4, 103.18) = 20.97	<0.001 ***	e > a, b, c d > a, b c > a
Elementary school ^b^	90 (35)	6.47 ± 5.48				123.72 ± 23.31			
Middle school ^c^	44 (17.1)	7.79 ± 5.30				131.20 ± 25.18			
High school ^d^	52 (20.2)	10.51 ± 5.30				138.59 ± 22.41			
University ^e^	47 (18.3)	13.95 ± 3.60				148.04 ± 20.43			
Income status									
Very poor	61 (23.7)	8.45 ± 5.72	F = 2.029	0.134		130.55 ± 25.02	F = 0.852	0.428	
Poor	94 (36.6)	7.91 ± 5.84				129.50 ± 24.14			
Moderate + Good	102 (39.7)	9.55 ± 5.81				133.91 ± 24.53			
Marital status									
Married ^a^	174 (67.7)	7.54 ± 5.87	Welch F (2, 102.35) = 68.74	<0.001 ***	b > a, c	127.90 ± 24.74	Welch F (2, 85.98) = 21.51	<0.001 ***	b > a, c
Single ^b^	38 (14.8)	13.92 ± 2.13				148.78 ± 16.10			
Widowed/divorced ^c^	45 (17.5)	8.75 ± 5.39				130.80 ± 23.57			
Employment status									
Employed	74 (28.8)	10.51 ± 5.70	t = 3.237	<0.001 ***		137.09 ± 24.46	t = 2.348	0.020 *	
Not employed	183 (71.2)	7.96 ± 5.72				129.24 ± 24.20			
Health assessment									
Very poor ^a^	19 (7.4)	6.73 ± 5.34	F = 2.839	0.039 *	d > a, b	123.89 ± 23.95	F = 1.824	0.143	
Poor ^b^	38 (14.8)	6.84 ± 5.35				125.42 ± 23.28			
Average ^c^	113 (44)	8.94 ± 6.08				133.77 ± 25.75			
Good + Very good ^d^	87 (33.9)	9.60 ± 5.58				132.86 ± 23.04			
Presence of chronic disease									
Yes	103 (40.1)	6.98 ± 5.75	t = −3.970	<0.001 ***		125.18 ± 24.43	t = −3.453	<0.001 ***	
No	154 (59.9)	9.84 ± 5.60				135.72 ± 23.68			
Regular medication use									
Yes	110 (42.8)	7.39 ± 5.71	t = −3.161	0.002 **		127.54 ± 25.38	t = −2.258	0.025 *	
No	147 (57.2)	9.67 ± 5.73				134.46 ± 23.46			
History of infectious disease after the earthquake									
Yes	159 (61.9)	8.28 ± 5.90	t = −1.430	0.154		129.52 ± 24.47	t = −1.656	0.099	
No	98 (38.1)	9.35 ± 5.67				134.71 ± 24.31			
Willingness to participate in communicable disease education									
Yes	176 (68.5)	6.71 ± 5.74	t = −11.528	<0.001 ***		123.09 ± 23.12	t = −10.705	<0.001 ***	
No	81 (31.5)	13.01 ± 3.00				149.76 ± 16.01			

IDKQ = Infectious Diseases Knowledge Questionnaire. CDRAPS = Communicable Diseases Risk Awareness and Protection Scale. M ± SD = Mean ± Standard Deviation. * *p* < 0.05, ** *p* < 0.01, *** *p* < 0.001. t = Independent samples *t*-test; F = One-way ANOVA with Tukey HSD post hoc test; Welch F = Welch’s ANOVA with Games–Howell post hoc test. “For age, a = 18–44 years, b = 45–64 years, and c = ≥65 years. For education status, a = literate, b = elementary school, c = middle school, d = high school, and e = university. For marital status, a = married, b = single, and c = widowed/divorced. For health assessment, a = very poor, b = poor, c = average, and d = good/very good.”.

**Table 2:** Mean scores and correlation coefficients for IDKQ and CDRAPS.

Scales	Min–Max	M ± SD	1	2	3	4	5	6	7
IDKQ Total (1)	0–17	8.69 ± 5.82							
Common Life Risk Awareness (2)	17–45	31.82 ± 5.74	0.729 ***						
Personal Protection Awareness (3)	17–40	30.01 ± 5.40	0.835 ***	0.836 ***					
Protection Behaviors (4)	16–40	28.46 ± 7.82	0.896 ***	0.775 ***	0.904 ***				
Hand Washing Behaviors (5)	6–15	11.63 ± 2.41	0.843 ***	0.751 ***	0.878 ***	0.926 ***			
Social Protection Awareness (6)	8–20	14.36 ± 2.33	0.481 ***	0.618 ***	0.608 ***	0.556 ***	0.537 ***		
Personal Contact Awareness (7)	6–20	15.22 ± 3.11	0.843 ***	0.770 ***	0.876 ***	0.887 ***	0.903 ***	0.527 ***	
CDRAPS Total (8)	82–180	131.50 ± 24.49	0.877 ***	0.897 ***	0.96 ***	0.957 ***	0.93 ***	0.671 ***	0.923 ***

Min–Max = Minimum–Maximum; M ± SD = Mean ± Standard Deviation. IDKQ = Infectious Diseases Knowledge Questionnaire; CDRAPS = Communicable Diseases Risk Awareness and Protection Scale. *** *p* < 0.001.

**Table 3:** Parallel mediation model examining the effects of awareness subdimensions on the relationship between infectious disease knowledge and protection and handwashing behaviors.

Variables		Protection Behaviors (Y_1_)					95% CI	
		Unstandardized Coefficients		The Standardized Coefficient	t	p	LLCI	ULCI
		B	SE	β				
Model_1_	Constant	−1.876	1.474	—	−1.272	0.204	−4.779	1.027
	IDKQ (X)	0.520	0.056	0.388	9.320	0.000	0.410	0.630
	Common Life Risk Awareness (M_1_)	−0.020	0.054	−0.015	−0.376	0.707	−0.126	0.086
	Self-Protection Awareness (M_2_)	0.542	0.078	0.374	6.935	0.000	0.388	0.696
	Social Protection Awareness (M_3_)	0.107	0.090	0.032	1.178	0.240	−0.072	0.285
	Personal Contact Awareness (M_4_)	0.568	0.119	0.226	4.763	0.000	0.333	0.803
	R^2^	0.893						
	F	417.633						
	p	0.000						
**Variables**		**Handwashing Behaviors (Y_2_)**					**95% CI**	
		**Unstandardized Coefficients**		**The Standardized Coefficient**	**t**	**p**	**LLCI**	**ULCI**
		**B**	**SE**	**β**				
Model_2_	Constant	1.166	0.526	—	2.219	0.027	0.131	2.201
	IDKQ (X)	0.077	0.020	0.187	3.892	0.000	0.038	0.117
	Common Life Risk Awareness (M_1_)	−0.013	0.019	−0.030	−0.667	0.505	−0.051	0.025
	Self-Protection Awareness (M_2_)	0.135	0.028	0.301	4.825	0.000	0.080	0.189
	Social Protection Awareness (M_3_)	0.024	0.032	0.024	0.758	0.449	−0.039	0.088
	Personal Contact Awareness (M_4_)	0.382	0.043	0.493	8.981	0.000	0.298	0.466
	R^2^	0.857						
	F	300.526						
	p	0.000						

IDKQ: Infectious Disease Knowledge Questionnaire; SE: Standard error; CI: Confidence Interval; LLCI: Lower Limit of 95% Confidence Interval; ULCI: Upper Limit of 95% Confidence Interval.

**Table 4:** Total, direct, and indirect effects of infectious disease knowledge on protection and handwashing behaviors through awareness subdimensions.

Model_1_	Effect	SE	t	*p*	95% CI	
					LLCI	ULCI
Total effect IDKQ → Protection Behaviors	1.202	0.037	32.215	0.000	1.129	1.276
Direct effect IDKQ → Protection Behaviors	0.520	0.056	9.320	0.000	0.410	0.630
Indirect Effect_1_ IDKQ → Common Life Risk Awareness → Protection Behaviors	−0.015	0.042	—	—	−0.106	0.059
Indirect Effect_2_ IDKQ → Self-Protection Awareness → Protection Behaviors	0.420	0.066	—	—	0.299	0.557
Indirect Effect_3_ IDKQ → Social Protection Awareness → Protection Behaviors	0.021	0.018	—	—	−0.013	0.058
Indirect Effect_4_ IDKQ → Personal Contact Awareness → Protection Behaviors	0.256	0.069	—	—	0.117	0.388
Total indirect effect	0.682	0.061	—	—	0.557	0.798
**Model_2_**	**Effect**	**SE**	**t**	***p***	**95% CI**	
					**LLCI**	**ULCI**
Total effect IDKQ → Handwashing Behaviors	0.349	0.014	25.045	0.000	0.322	0.377
Direct effect IDKQ → Handwashing Behaviors	0.077	0.020	3.892	0.000	0.038	0.117
Indirect Effect_5_ IDKQ → Common Life Risk Awareness → Handwashing Behaviors	−0.009	0.016	—	—	−0.041	0.023
Indirect Effect_6_ IDKQ → Self-Protection Awareness → Handwashing Behaviors	0.104	0.028	—	—	0.050	0.161
Indirect Effect_7_ IDKQ → Social Protection Awareness → Handwashing Behaviors	0.005	0.008	—	—	−0.010	0.021
Indirect Effect_8_ IDKQ → Personal Contact Awareness → Handwashing Behaviors	0.172	0.026	—	—	0.118	0.221
Total indirect effect	0.272	0.020	—	—	0.232	0.309

IDKQ: Infectious Disease Knowledge Questionnaire; SE: Standard error; CI: Confidence Interval; LLCI: Lower Limit of 95% Confidence Interval; ULCI: Upper Limit of 95% Confidence Interval.

## Data Availability

The data presented in this study are available on request from the corresponding author due to privacy.

## Supplementary Materials

The following supporting information can be downloaded at: https://www.mdpi.com/article/10.3390/healthcare14172688/s1, Table S1: Single-mediator models of awareness subdimensions in the relationship between infectious disease knowledge and protection and handwashing behaviors; Table S2: Total, direct, and indirect effects of infectious disease knowledge on protection and handwashing behaviors through individual awareness subdimensions; Figure S1: The mediating role of awareness sub-dimensions in the relationship between knowledge of infectious diseases and protection behaviors. * *p* < 0.05; ** *p* < 0.01; *** *p* < 0.001; Figure S2: The mediating role of awareness sub-dimensions in the relationship between knowledge of infectious diseases and hand-washing behaviors. * *p* < 0.05; ** *p* < 0.01; *** *p* < 0.001.
