# Supplementary material for: The Mediating Effect of Risk Awareness on the Relationship Between Infectious Disease Knowledge and Protective Behaviors Among Earthquake-Affected Populations
Source: Healthcare (Basel). 2026 Aug 24;14(17):2688. doi: 10.3390/healthcare14172688 (PMC13565647; doi:10.3390/healthcare14172688)
Supplement: Supplementary file 1 [file healthcare-14-02688-s001.zip › healthcare-4447197-supplementary.pdf]

Supplementary Table S1. Single-mediator models of awareness subdimensions in the relationship between infectious disease knowledge and protection and handwashing behaviors

**Variables**		**Protection Behaviors (Y_1)**					**95% CI**	
		**Unstandardized Coefficients**		**The standardized coefficient**	***t***	***p***	**LLCI**	**ULCI**
		B	***SE***	***β***				
Model$_1$	Constant	8.965	1.350	–	6.641	0.000	6.307	11.624
	IDKQ (X)	0.948	0.050	0.707	18.935	0.000	0.850	1.047
	Common Life Risk Awareness (M_1)	0.353	0.051	0.259	6.946	0.000	0.253	0.454
	R^2	0.834						
	F	639.174						
	p	0.000						

Variables		Protection Behaviors (Y_1)					95% CI	
		Unstandardized Coefficients		The standardized coefficient	*t*	*p*	LLCI	ULCI
		B	*SE*	*β*				
Model$_2$	Constant	0.686	1.355	–	0.506	0.613	-1.982	3.354
	IDKQ (X)	0.627	0.053	0.467	11.926	0.000	0.523	0.730
	Self-Protection Awareness (M_2)	0.744	0.057	0.514	13.115	0.000	0.632	0.856
	R^2	0.882						
	F	952.875						
	p	0.000						

Variables		Protection Behaviors (Y_1)					95% CI	
		Unstandardized Coefficients		The standardized coefficient				
		B	*SE*	*β*	*t*	*p*	LLCI	ULCI
Model$_3$	Constant	11.092	1.331	–	8.331	0.000	8.470	13.713
	IDKQ (X)	1.097	0.040	0.818	27.173	0.000	1.018	1.177
	Social Protection Awareness (M_3)	0.545	0.101	0.163	5.406	0.000	0.346	0.743
	R^2	0.823						
	F	590.950						
	p	0.000						

Variables		Protection Behaviors (Y_1)					95% CI	
		Unstandardized Coefficients		The standardized coefficient				
		B	*SE*	*β*	*t*	*p*	LLCI	ULCI
Model$_4$	Constant	5.131	1.271	–	4.038	0.000	2.628	7.634
	IDKQ (X)	0.689	0.058	0.513	11.855	0.000	0.574	0.803
	Personal Contagion Awareness (M_4)	1.139	0.109	0.454	10.483	0.000	0.925	1.353
	R^2	0.862						
	F	795.445						
	p	0.000						

Variables		Handwashing Behaviors (Y_2)					95% CI	
		Unstandardized Coefficients		The standardized coefficient				
		B	*SE*	*β*	*t*	*p*	LLCI	ULCI
Model5	Constant	5.548	0.511	–	10.683	0.000	4.451	6.464
	IDKQ (X)	0.261	0.019	0.630	13.774	0.000	0.224	0.298
	Common Life Risk Awareness (M_1)	0.123	0.019	0.292	6.373	0.000	0.085	0.161
	R^2	0.866						
	F	382.653						
	p	0.000						

Variables		Handwashing Behaviors (Y_2)					95% CI	
		Unstandardized Coefficients		The standardized coefficient				
		B	*SE*	*β*	*t*	*p*	LLCI	ULCI
Model6	Constant	2.619	0.531	–	4.934	0.000	1.574	3.665
	IDKQ (X)	0.151	0.021	0.364	7.314	0.000	0.110	0.191
	Self-Protection Awareness (M_2)	0.123	0.022	0.574	11.552	0.000	0.213	0.301
	R^2	0.811						
	F	543.247						
	p	0.000						

Variables		Handwashing Behaviors (Y_2)					95% CI	
		Unstandardized Coefficients		The standardized coefficient				
		B	*SE*	*β*	*t*	*p*	LLCI	ULCI
Model7	Constant	6.347	0.504	–	12.583	0.000	5.354	7.340
	IDKQ (X)	0.315	0.015	0.761	20.598	0.000	0.285	0.345
	Social Protection Awareness (M_3)	0.177	0.038	0.172	4.645	0.000	0.102	0.253
	R^2	0.734						
	F	349.701						
	p	0.000						

Variables		Handwashing Behaviors (Y_2)					95% CI	
		Unstandardized Coefficients		The standardized coefficient				
		B	*SE*	*β*	*t*	*p*	LLCI	ULCI
Model8	Constant	2.774	0.424	–	6.536	0.000	1.938	3.610
	IDKQ (X)	0.117	0.019	0.282	6.030	0.000	0.079	0.155
	Personal Contagion Awareness (M_4)	0.515	0.036	0.665	14.197	0.000	0.444	0.587
	R^2	0.839						
	F	661.045						
	p	0.000						

IDKQ: Infections Disease Knowledge Questionnaire; SE: Standard error; CI: Confidence Interval; LLCI: Lower Limit of 95% Confidence Interval; ULCI: Upper Limit of 95% Confidence Interval

Supplementary Table S2. Total, direct, and indirect effects of infectious disease knowledge on protection and handwashing behaviors through individual awareness subdimensions

**Model$_1$**	**Effect**	***SE***	***t***	***p***	**95% CI**	
					**LLCI**	**ULCI**
Total effect<br>IDKQ→ Protection Behaviors	1.202	0.037	32.215	0.000	1.129	1.276
Direct effect<br>IDKQ→ Protection Behaviors	0.948	0.050	18.935	0.000	0.850	1.047
Indirect Effect$_1$<br>IDKQ→Common Life Risk Awareness→Protection Behaviors	0.254	0.044	–	–	0.170	0.345
**Model$_2$**	Effect	*SE*	*t*	*p*	95% CI	
					LLCI	ULCI
Total effect<br>IDKQ→ Protection Behaviors	1.202	0.037	32.215	0.000	1.129	1.276
Direct effect<br>IDKQ→ Protection Behaviors	0.627	0.053	11.926	0.000	0.523	0.730
Indirect Effect$_2$<br>IDKQ→Self-Protection Awareness→Protection Behaviors	0.576	0.047	–	–	0.483	0.669
**Model$_3$**	Effect	*SE*	*t*	*p*	95% CI	
					LLCI	ULCI
Total effect<br>IDKQ→ Protection Behaviors	1.202	0.037	32.215	0.000	1.129	1.276
Direct effect<br>IDKQ→ Protection Behaviors	1.097	0.040	27.173	0.000	1.018	1.177
Indirect Effect$_3$<br>IDKQ→Social Protection Awareness→Protection Behaviors	0.105	0.026	–	–	0.059	0.159
**Model$_4$**	Effect	*SE*	*t*	*p*	95% CI	
					LLCI	ULCI
Total effect<br>IDKQ→ Protection Behaviors	1.202	0.037	32.215	0.000	1.129	1.276
Direct effect<br>IDKQ→ Protection Behaviors	0.689	0.058	11.855	0.000	0.574	0.803
Indirect Effect$_4$<br>IDKQ→Personal Contagion Awareness→Protection Behaviors	0.513	0.065	–	–	0.387	0.640
**Model$_5$**	Effect	*SE*	*t*	*p*	95% CI	
					LLCI	ULCI
Total effect<br>IDKQ→ Handwashing Behaviors	0.349	0.014	25.045	0.000	0.322	0.377
Direct effect<br>IDKQ→ Handwashing Behaviors	0.261	0.019	13.774	0.000	0.224	0.298
Indirect Effect<br>IDKQ→Common Life Risk Awareness→Handwashing Behaviors	0.088	0.019	–	–	0.054	0.127
**Model$_6$**	Effect	*SE*	*t*	*p*	95% CI	
					LLCI	ULCI
Total effect<br>IDKQ→ Handwashing Behaviors	0.349	0.014	25.045	0.000	0.322	0.377
Direct effect<br>IDKQ→ Handwashing Behaviors	0.151	0.021	7.314	0.000	0.110	0.191
Indirect Effect<br>IDKQ→Self-Protection Awareness→Handwashing Behaviors	0.199	0.021	–	–	0.157	0.238
**Model$_7$**	Effect	*SE*	*t*	*p*	95% CI	
					LLCI	ULCI

Total effect<br>IDKQ→ Handwashing Behaviors	0.349	0.014	25.045	0.000	0.322	0.377
Direct effect<br>IDKQ→ Handwashing Behaviors	0.315	0.015	20.598	0.000	0.285	0.345
Indirect Effect<br>IDKQ→Social Protection Awareness→Handwashing Behaviors	0.034	0.010	–	–	0.016	0.056
**Model**$_8$	Effect	*SE*	*t*	*p*	95% CI	
					LLCI	ULCI
Total effect<br>IDKQ→ Handwashing Behaviors	0.349	0.014	25.045	0.000	0.322	0.377
Direct effect<br>IDKQ→ Handwashing Behaviors	0.117	0.019	6.030	0.000	0.079	0.155
Indirect Effect<br>IDKQ→Personal Contagion Awareness→Handwashing Behaviors	0.232	0.020	–	–	0.192	0.272

IDKQ: Infections Disease Knowledge Questionnaire; SE: Standard error; CI: Confidence Interval; LLCI: Lower Limit of 95% Confidence Interval; ULCI: Upper Limit of 95% Confidence Interval

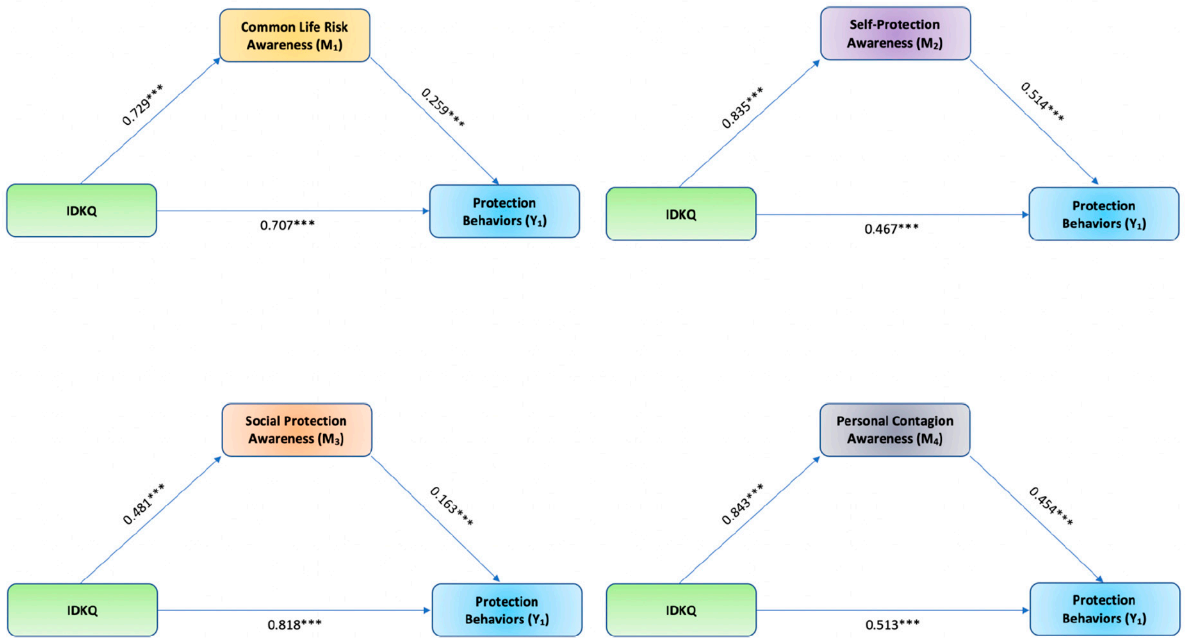

Supplementary Figure S1. The mediating role of awareness sub-dimensions in the relationship between knowledge of infectious diseases and protection behaviors. $^{*}p < 0.05$; $^{**}p < 0.01$; $^{***}p < 0.001$.

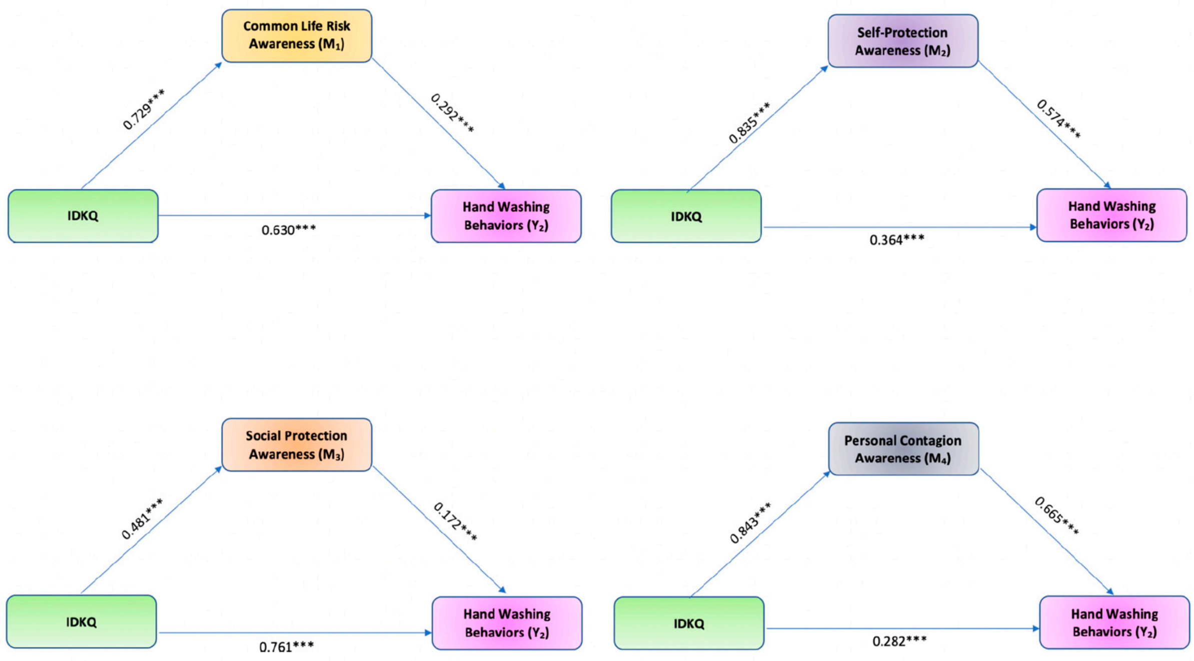

Supplementary Figure S2. The mediating role of awareness sub-dimensions in the relationship between knowledge of infectious diseases and hand-washing behaviors. *p < 0.05; **p < 0.01; ***p < 0.001.

Single-Mediation Analysis of Awareness Subdimensions

Supplementary Table S1 presents the effects of infectious disease knowledge and the awareness subdimensions on protection behaviors and handwashing behaviors. For protection behaviors (Y_1), Model 1 examined the effects of infectious disease knowledge (IDKQ) and Common Life Risk Awareness (M_1). The results showed that both IDKQ ($\beta = 0.707$, $p < 0.001$) and Common Life Risk Awareness ($\beta = 0.259$, $p < 0.001$) were significant positive predictors of protection behaviors. The model was statistically significant ($F = 639.174$, $p < 0.001$) and explained 83.4% of the variance in protection behaviors ($R^2 = 0.834$).

Model 2 assessed the effects of IDKQ and Self-Protection Awareness (M_2) on protection behaviors. Both IDKQ ($\beta = 0.467$, $p < 0.001$) and Self-Protection Awareness ($\beta = 0.514$, $p < 0.001$) significantly and positively predicted protection behaviors. The model was statistically significant ($F = 952.875$, $p < 0.001$) and accounted for 88.2% of the variance in protection behaviors ($R^2 = 0.882$).

Model 3 investigated the effects of IDKQ and Social Protection Awareness (M_3) on protection behaviors. IDKQ remained a significant positive predictor ($\beta = 0.818$, $p < 0.001$), while Social Protection Awareness also demonstrated a significant positive effect ($\beta = 0.163$, $p < 0.001$). The model explained 82.3% of the variance in protection behaviors ($R^2 = 0.823$) and was statistically significant ($F = 590.950$, $p < 0.001$).

Model 4 evaluated the effects of IDKQ and Personal Contagion Awareness (M_4) on protection behaviors. Both IDKQ ($\beta = 0.513$, $p < 0.001$) and Personal Contagion Awareness ($\beta = 0.454$, $p < 0.001$) were significant positive

predictors of protection behaviors. The model was statistically significant ($F = 795.445$, $p < 0.001$) and explained 86.2% of the variance in protection behaviors ($R^2 = 0.862$).

For handwashing behaviors (Y_2), Model 5 examined the effects of IDKQ and Common Life Risk Awareness. Both IDKQ ($\beta = 0.630$, $p < 0.001$) and Common Life Risk Awareness ($\beta = 0.292$, $p < 0.001$) significantly and positively predicted handwashing behaviors. The model explained 86.6% of the variance in handwashing behaviors ($R^2 = 0.866$) and was statistically significant ($F = 382.653$, $p < 0.001$).

Model 6 assessed the effects of IDKQ and Self-Protection Awareness on handwashing behaviors. Both predictors were significant, with Self-Protection Awareness ($\beta = 0.574$, $p < 0.001$) showing a stronger effect than IDKQ ($\beta = 0.364$, $p < 0.001$). The model explained 81.1% of the variance in handwashing behaviors ($R^2 = 0.811$) and was statistically significant ($F = 543.247$, $p < 0.001$).

Model 7 investigated the effects of IDKQ and Social Protection Awareness on handwashing behaviors. IDKQ ($\beta = 0.761$, $p < 0.001$) and Social Protection Awareness ($\beta = 0.172$, $p < 0.001$) were both significant positive predictors. The model was statistically significant ($F = 349.701$, $p < 0.001$) and accounted for 73.4% of the variance in handwashing behaviors ($R^2 = 0.734$).

Model 8 examined the effects of IDKQ and Personal Contagion Awareness on handwashing behaviors. Both IDKQ ($\beta = 0.282$, $p < 0.001$) and Personal Contagion Awareness ($\beta = 0.665$, $p < 0.001$) significantly predicted handwashing behaviors. The model explained 83.9% of the variance in handwashing behaviors ($R^2 = 0.839$) and was statistically significant ($F = 661.045$, $p < 0.001$).

Overall, infectious disease knowledge was a significant positive predictor of both protection behaviors and handwashing behaviors across all models. In addition, all awareness subdimensions were positively associated with both behavioral outcomes. Among the awareness subdimensions, Self-Protection Awareness and Personal Contagion Awareness demonstrated the strongest associations with protection and handwashing behaviors, respectively.

As shown in Supplementary Table S2 and Figure a and b, the mediating roles of the awareness subdimensions in the relationship between infectious disease knowledge and protection behaviors as well as handwashing behaviors were examined using bootstrap mediation analyses.

For protection behaviors (Y_1), the total effect of infectious disease knowledge on protection behaviors was significant ($B = 1.202$, $p < 0.001$). In Model 1, Common Life Risk Awareness significantly mediated the relationship between infectious disease knowledge and protection behaviors. Although the direct effect remained significant ($B = 0.948$, $p < 0.001$), the indirect effect through Common Life Risk Awareness was also significant ($B = 0.254$, 95% CI = 0.170–0.345), indicating partial mediation. In Model 2, Self-Protection Awareness demonstrated a significant mediating effect ($B = 0.576$, 95% CI = 0.483–0.669). The direct effect remained significant ($B = 0.627$, $p < 0.001$), suggesting partial mediation. In Model 3, Social Protection Awareness also significantly mediated the relationship between infectious disease knowledge and protection behaviors ($B = 0.105$, 95% CI = 0.059–0.159), while the direct effect remained significant ($B = 1.097$, $p < 0.001$). Similarly, in Model

4, Personal Contagion Awareness exerted a significant indirect effect (B = 0.513, 95% CI = 0.387–0.640), whereas the direct effect of infectious disease knowledge on protection behaviors remained significant (B = 0.689, $p < 0.001$). Among the awareness subdimensions, Self-Protection Awareness showed the strongest indirect effect, followed by Personal Contagion Awareness.

For handwashing behaviors (Y_2), the total effect of infectious disease knowledge was also significant (B = 0.349, $p < 0.001$). In Model 5, Common Life Risk Awareness significantly mediated the relationship between infectious disease knowledge and handwashing behaviors (B = 0.088, 95% CI = 0.054–0.127), while the direct effect remained significant (B = 0.261, $p < 0.001$). In Model 6, Self-Protection Awareness demonstrated a significant indirect effect (B = 0.199, 95% CI = 0.157–0.238), indicating partial mediation. The direct effect remained significant (B = 0.151, $p < 0.001$). In Model 7, Social Protection Awareness also showed a significant indirect effect (B = 0.034, 95% CI = 0.016–0.056), although its mediating effect was relatively small compared with the other awareness dimensions. The direct effect remained significant (B = 0.315, $p < 0.001$). Finally, in Model 8, Personal Contagion Awareness significantly mediated the relationship between infectious disease knowledge and handwashing behaviors (B = 0.232, 95% CI = 0.192–0.272), while the direct effect remained significant (B = 0.117, $p < 0.001$).

Overall, none of the bootstrap confidence intervals for the indirect effects included zero, indicating that all awareness subdimensions significantly mediated the relationship between infectious disease knowledge and both protection behaviors and handwashing behaviors. Because the direct effects remained statistically significant in all models, the findings support partial mediation. Self-Protection Awareness exhibited the strongest mediating effect for protection behaviors, whereas Personal Contagion Awareness demonstrated the strongest mediating effect for handwashing behaviors.
